# Assessment of obsessive and compulsive symptoms in patients with schizophrenia

**DOI:** 10.1192/j.eurpsy.2022.1652

**Published:** 2022-09-01

**Authors:** S. Ajmi, S. Ellouze, M. Abdelkefi, M. Turki, N. Halouani, J. Aloulou

**Affiliations:** 1 CHU Hedi Chaker, Psychiatry, sfax, Tunisia; 2 Hedi Chaker University Hospital, Psychiatry B, Sfax, Tunisia; 3 Hedi Chaker University Hospital, Psychiatry “b” Department, Sfax, Tunisia; 4 Hedi Chaker University Hospital, Psychiatry, Sfax, Tunisia

**Keywords:** Obsessive Compulsive Symptoms, Treatment, schizophrénia, comorbidity

## Abstract

**Introduction:**

Obsessive-Compulsive Symptoms (OCS) are common in patients with schizophrenia, with a prevalence of 3.5% to 25%.

**Objectives:**

The aim of our study was to assess the frequency of OCS in patients with schizophrenia, and to study the clinical and evolutionary characteristics of schizophrenia and OCS comorbidity.

**Methods:**

We conducted a cross-sectional, descriptive, and analytical study. Thirty schizophrenic patients were recruited in the department of psychiatry B of Hedi Chaker university hospital of Sfax. We used the Yale-Brown Obsession-Compulsion Scale (Y-BOCS) to assess obsessive and compulsive symptoms, at the end of hospitalization, after clinical remission of schizophrenic symptoms.

**Results:**

The mean age of patients was 41.2, that of disease onset was 27.3. Most of patients were male (86.7%) and unemployed (81.3%). A personal history of suicide attempts was found in 16.6% of patients. The average number of hospitalizations was 8.83. OCS were noted in 36% of patients with a Y-BOCS mean score of 5.5. Patients with OCS had significantly more frequent alcohol use (p = 0,008), a higher number (p = 0.03) and longer duration of hospitalizations (P = 0,034) and are more frequently treated with atypical antipsychotics (p = 0.001).

**Conclusions:**

Our results show that patients with schizophrenia frequently present OCS. This comorbidity has a negative impact on the evolution and the prognosis of the disease, as well as the functioning of patients. Therefore, it should be investigated in order to ensure better care and promote the socio-professional reintegration of these patients.

**Disclosure:**

No significant relationships.

